# Pediatric hospital admissions, case severity, and length of hospital stay during the first 18 months of the COVID-19 pandemic in a tertiary children’s hospital in Switzerland

**DOI:** 10.1007/s15010-022-01911-x

**Published:** 2022-09-05

**Authors:** Jasmin Bögli, Sabine Güsewell, Rita Strässle, Christian R. Kahlert, Werner C. Albrich

**Affiliations:** 1 Division of Infectious Diseases and Hospital Epidemiology, Cantonal Hospital St. Gallen, Rorschacherstrasse 95, 9007 St. Gallen, Switzerland; 2 Department of Infectious Diseases and Hospital Epidemiology, Children’s Hospital of Eastern Switzerland, Claudiusstr. 6, 9006 St. Gallen, Switzerland; 3 University of Zurich, Zurich, Switzerland; 4 University of St. Gallen, St. Gallen, Switzerland

**Keywords:** SARS-CoV-2, Pandemics, Hospital admission rates, Respiratory infections, Non-respiratory infections

## Abstract

**Background:**

SARS-CoV-2 directly contributes to the burden of respiratory disease in children, but indirect effects of protective measures also need to be considered to assess the overall impact of the pandemic on children's health.

**Methods:**

We retrospectively compared pre-pandemic and pandemic data of main admission diagnoses, sorted by ICD-10 diagnosis groups, in a tertiary children's hospital in Switzerland from 2017 until August 2021. Hospital admission rates, severity, and length of stay (LOS) of the individual ICD-10 groups during the pandemic were compared with three previous years accounting for seasonal differences.

**Results:**

Among 20,168 hospital admissions *(n* = *13′950 in pre-pandemic years; n* = *3′120 in 2020 and n* = *3′098 in 2021)*, there were significant decreases in numbers of admissions for respiratory diseases during the early pandemic with a rebound in summer 2021. During the pandemic, admissions for non-respiratory infections, neoplasms, and skin diseases decreased but increased for trauma. Particularly, a drop in admissions for different respiratory infections [e.g. respiratory syncytial virus (RSV) and bronchiolitis] was pronounced after introduction of strict measures, but admissions increased again after restrictions were loosened. While disease severity was lower for respiratory and neurologic diseases and bronchiolitis throughout the pandemic, gastrointestinal disease admissions had longer LOS and in the first pandemic year greater severity. For RSV and pneumonia, disease severity and LOS were higher in the first pandemic year and lower in the second pandemic year.

**Conclusion:**

The pandemic and associated protective measures had a significant effect on respiratory and non-respiratory admissions, particularly with decreases in hospital admissions for respiratory infections followed by a rebound after loosening of measures.

**Supplementary Information:**

The online version contains supplementary material available at 10.1007/s15010-022-01911-x.

## Introduction

The effect of the current SARS-CoV-2 pandemic including consequences of both virus and protective measures on child health is still under debate. During the first lockdown in Switzerland in spring 2020, public and social life was severely restricted [1, 2]. Subsequently, restrictions were partially lifted depending on transmission intensity and hospital occupancy.

While contact restrictions are effective against communicable diseases, different patterns of recreational activities may also have an impact on healthcare-seeking behaviors [3]. This led us to the hypothesis that the pandemic and associated measures impacted a wide range of childhood diseases far beyond respiratory diseases. Previous studies examining hospital admission rates during the pandemic mainly focused on the first pandemic year and were restricted to a few non-COVID-19 medical conditions only [4–18].

The aim of this study was to compare the frequency of diagnoses, severity of cases, and length of stay (LOS) in children admitted to our tertiary care children’s hospital during the first 18 months of the SARS-CoV-2 pandemic with pre-pandemic periods.

## Patients & methods

The ICD-10 codes of main discharge diagnoses from all hospital admissions (except for rare diagnoses of < 52 admissions/year) of children (≤ 18 years of age) at the largest non-university hospital in Switzerland from 21 March 2020 to 31 August 2021 (defined as pandemic) and from 21 March 2017 to 20 March 2020 (defined as pre-pandemic and reference period) were obtained retrospectively from the hospital’s coding office. Identified cases were included in the analysis.

Disease severity and LOS among all inpatients were investigated. Case severity was evaluated using the patient clinical complexity level (PCCL; score from 0 to 6 calculated from several severity-weighted secondary diagnoses (CCL values) of a patient [19].

### Pandemic stages in Switzerland

In Switzerland, the following 6 phases of the COVID-19 pandemic can be distinguished (Suppl. Figure 1A, 1B):

1. First national comprehensive lockdown phase [only phase with closures of compulsory schools and secondary schools (high school and vocational education), hospitals suspended non-urgent elective surgery and outpatient visits]: 16.3.2020–26.4.2020
2. Reopening phase from first lockdown: 27.4.2020–6.6.2020
3. Few restrictions: 7.6.2020–17.10.2020
4. Second national lockdown: 18.10.2020–29.2.2021
5. Reopening phase from second lockdown: 1.3.2021–26.6.2021
6. Few restrictions: 27.6.2021–13.9.2021.

### Statistical analysis

Season-specific changes in weekly numbers of hospital admissions by ICD-10 diagnosis groups (Table 1) during either year of the pandemic relative to the reference period were modeled with Poisson regression including effects of COVID-19, season, and their interaction. Years 1–3 (21 March 2017 until 20 March 2020) were reference period (“pre-pandemic”), year 4 (21 March 2020—21 March 2021) first year of the COVID-19 pandemic, and year 5 (21 March—31 August 2021) the first 5.3 months of the second pandemic year. Because numbers dropped to 0 during an entire season for some of the diagnoses, Bayesian models were fitted. Mean weekly numbers of admissions in each season were obtained as model predictions. Rate ratios for changes in weekly admissions due to COVID-19 in each season were obtained from the model coefficients of separate models fitted without intercept. Note that our study was explorative. Although we present p values for group differences, they should be considered descriptive and not as formal tests of significance. Accordingly, we did not perform multiple comparisons, nor did we correct for multiple testing.

**Table 1:** Mean weekly number of admissions in each season before the pandemic (B, from 21 March 2017 to 20 March 2020) in the first year (P1, from 21 March 2020 to 20 March 2021), and in the second year (P2, from 21 March until 31 August 2021) of the pandemic

	Spring			Summer			Autumn		Winter	
	B	P 1	P 2	B	P 1	P 2	Before	P1	Before	P1
RSV	1.2	0.3	0.2	0.1	0.0	13.5	0.8	0.0	9.0	0.0
Bronchiolitis	1.5	0.2	0.8	0.2	0.2	8.0	1.1	0.1	8.0	0.3
Pneumonia	1.9	0.7	0.9	1.1	0.3	3.2	1.7	0.4	5.4	0.6
Influenza	0.1	0.1	0.0	0.0	0.0	0.0	0.1	0.0	3.1	0.0
Otitis	2.3	0.6	1.6	1.2	0.5	1.9	1.5	0.6	3.9	0.6
Mastoiditis	0.2	0.2	0.1	0.3	0.1	0.2	0.1	0.3	0.2	0.0
Pertussis	0.0	0.0	0.0	0.1	0.0	0.0	0.0	0.0	0.1	0.0
Preterm birth	3.0	1.9	1.5	2.5	1.5	1.7	2.5	2.5	2.7	1.9

"0.0" is the rounded value for fitted mean numbers < 0.05. Note that spring and summer seasons were defined slightly differently in P1 and P2. For the sake of simplicity, only means of the full seasons (as in P1) are reported for the pre-pandemic years (B) in this table

Case severity was analyzed by plotting the proportion of severity grades (from 0 to 6) for the reference period, the first year of the pandemic, and the second year until 31 August, and by calculating the proportion of severe cases (grades 3–6) for these periods. LOS was analyzed by plotting the distribution of durations (days) for the reference period, the first year of the pandemic, and the second year until 31 August, and by calculating the median duration for the three periods. Shifts in severity grades or durations toward higher or lower values between the three periods were tested with Kruskal–Wallis tests.

## Results

### Hospital admission rates

**Fig. 1:**
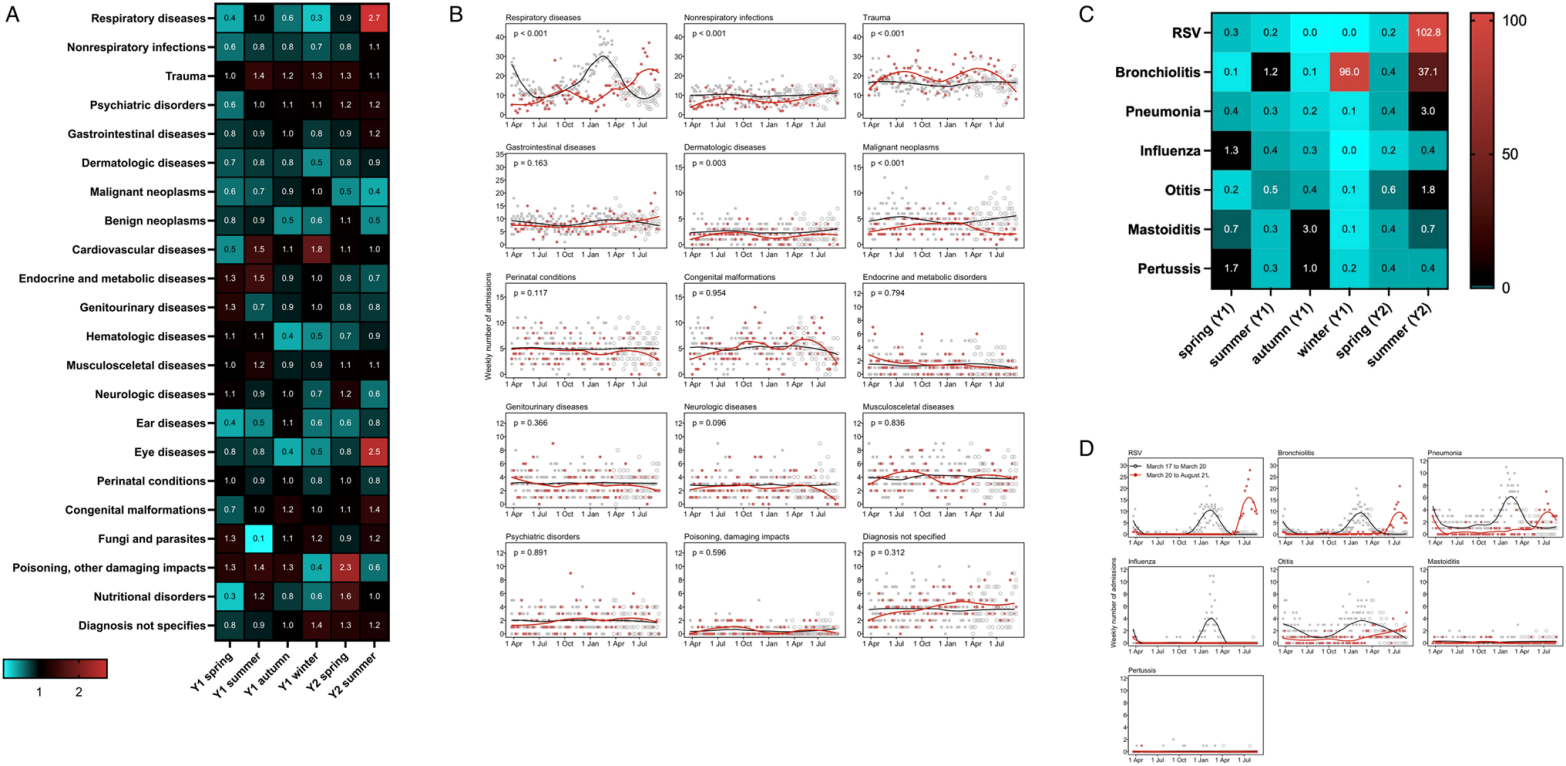
**A** Mean number of hospital admissions during pre-pandemic and pandemic years for major diagnostic groups. For more detailed information including the rate ratios for changes over time, see suppl. Table 2. **B** Admissions during pre-pandemic and pandemic years for major diagnostic groups. Weekly number of admissions from 21 March 2017 to 31 August 2021 (points) and smoothing lines obtained with local polynomial regression (loess) separately for the 3 years preceding the pandemic and during the pandemic. To represent the entire time course of the pandemic by one line, the time axis ranges from 21 March 2020 to 31 August 2021. For the 3 years preceding the pandemic, weeks from 21 March to 31 August are duplicated at the end of the time axis (open symbols). P values from Poisson models for the overall difference between the two periods (regardless of season) are given. **C** Mean number of hospital admissions during pre-pandemic and pandemic years. For more detailed information including the rate ratios for changes over time, see Table 2. **D** Admissions during pre-pandemic and pandemic years. Weekly number of admissions from 21 March 2017 to 31 August 2021 (points) and smoothing lines obtained with local polynomial regression (loess) separately for the 3 years preceding the pandemic and during the pandemic. To represent the entire time course of the pandemic (to date), the time axis ranges from 21 March to 31 August. For the three years preceding the pandemic, weeks from 21 March to 31 August are duplicated at the end of the time axis (open symbols), whereas the true time course is shown for the pandemic (21 March 2020 to 31 August 2021)

Overall, 20′168 hospital admissions (*n* = 13′950 in pre-pandemic years; *n* = 3′120 in 2020 and *n* = 3′098 in 2021) were included in the analysis. This represented 97.4% of all admissions, while rare diseases were excluded. There was a decrease in the overall occupancy rate of the Children's Hospital of Eastern Switzerland during the pandemic in most months (Suppl. Figure 2). Comparing the pandemic period with the reference period, the rate ratios for the following ICD-10 diagnosis groups showed significant differences depending on the season (Suppl. Table 2, Fig. 1A): respiratory diseases (decreases in in spring [rate ratio: 0.4], in autumn [RR: 0.6], and winter [RR: 0.3] 2020; increases in summer 2021 [RR: 2.7]), non-respiratory infections (decreases in spring [RR: 0.6], in summer [RR: 0.8] and in winter [RR: 0.7] 2020), trauma (increases in summer [RR: 1.4] and in winter [RR: 1.3] 2020 and in spring [RR: 1.3] 2021). For the following ICD-10 diagnosis groups, significant decreases were found over the entire period without seasonal influence (Fig. 1B, Suppl. Figure 3A): dermatologic diseases (*p = *0.003), malignant (*p < *0.001), or benign (*p = *0.015) neoplasms. For the remaining ICD-10 diagnosis groups, hospital admission rate ratios remained unchanged.

The mean numbers of hospital admissions were significantly lower during the pandemic in almost all seasons during the first pandemic year for the diagnoses RSV (*p < *0.001 in both the first and the second pandemic years), pneumonia (*p < *0.001 in first pandemic year), otitis (*p < *0.001 in first pandemic year), and bronchiolitis (*p < *0.001 in both the first and the second pandemic years) (Suppl. Table 3 and Table 2, Fig. 1C, D). Influenza showed a significant decrease in the mean number of admissions to 0.0 during winter 2020 (Table 2, Fig. 1D). In contrast, during summer of the second pandemic year, there were significant increases in hospital admission rates for respiratory syncytial virus (RSV) (RR: 102.8), bronchiolitis (RR: 37.1), pneumonia (RR: 3.0), and otitis (RR: 1.8; Table 2, Fig. 1D) but not for influenza. For most of the separately evaluated respiratory diagnoses, there were both seasonal and pandemic effects on number of hospital admissions (Table 2).

**Table 2:**
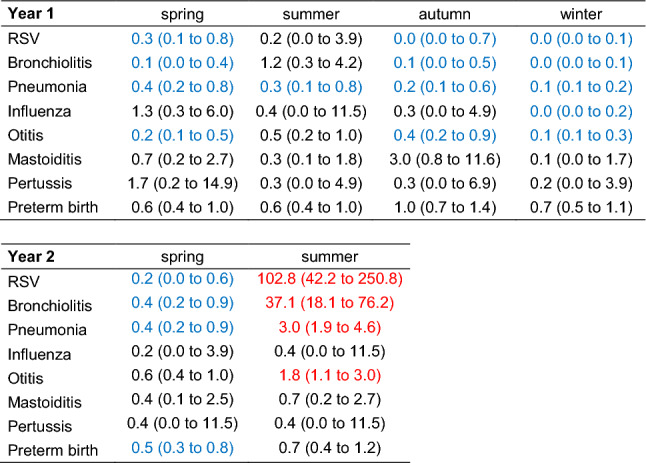
Rate ratios for changes in the mean weekly number of admissions in each season due to the pandemic

### Case severity and length of hospital stay

**Fig. 2:**
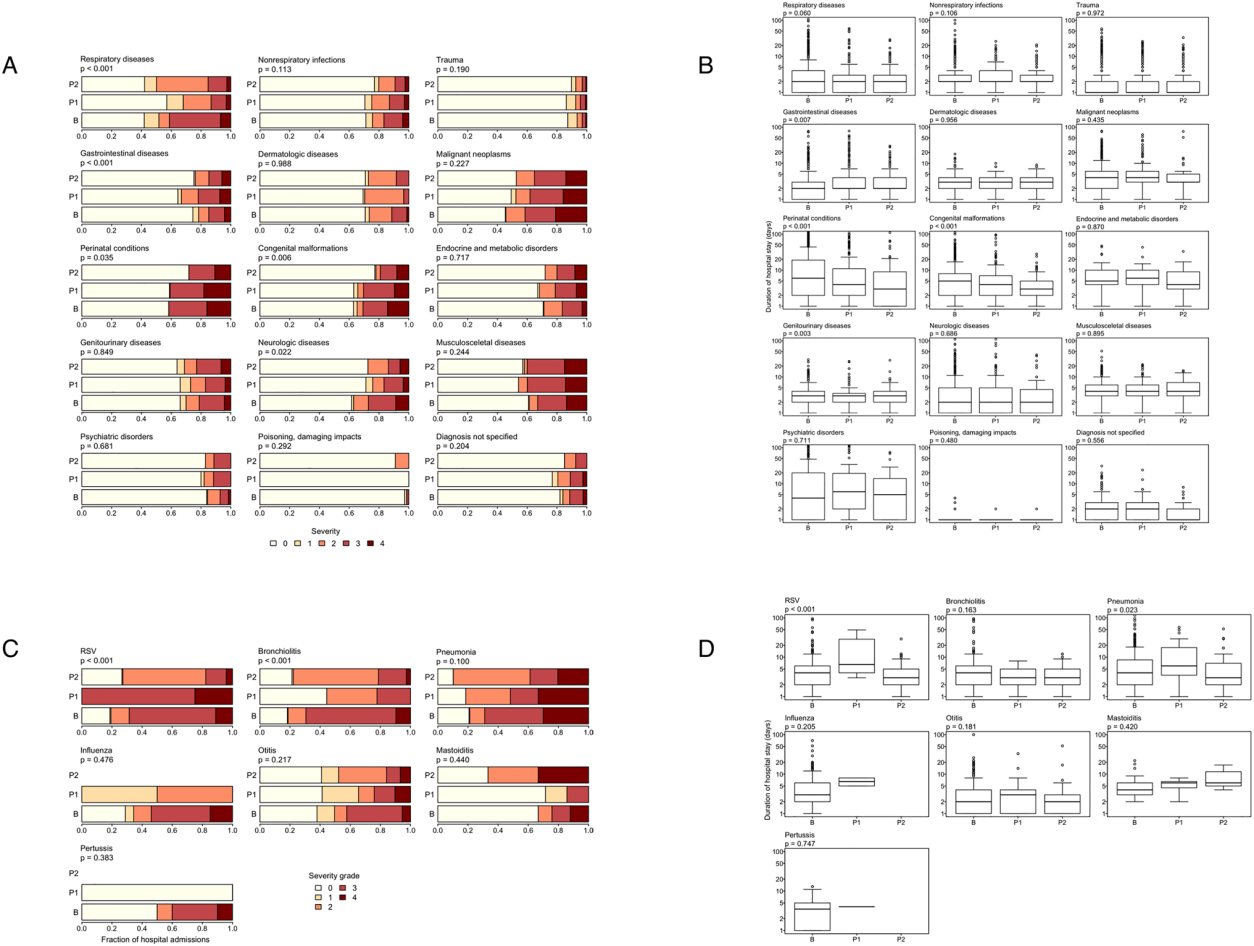
**A** Case severity during pre-pandemic and pandemic years for major diagnostic groups. Distribution of case severity (ranked from 0 to 4) among all cases admitted before the pandemic (B), during the first year of the pandemic (P1), and until August of the second year of the pandemic (P2) for each group of diagnoses. P values from Kruskal–Wallis rank sum tests comparing severity grades between the three periods are given. **B** Duration of hospital stay during pre-pandemic and pandemic years for major diagnostic groups. Distribution of the length of hospital stay among all cases admitted before the pandemic (B), during the first year of the pandemic (P1), and until August of the second year of the pandemic (P2) for each group of diagnoses. P values from Kruskal–Wallis rank sum tests comparing numbers of days between the three periods are given. **C** Case severity during pre-pandemic and pandemic years. Distribution of case severity (ranked from 0 to 4) among all cases admitted before the pandemic (B), during year 1 of the pandemic (P1), and until 31.08. of year 2 of the pandemic (P2), for each diagnosis. P values from Kruskal–Wallis rank sum tests comparing severity grades between the three periods are given. Note that some bars represent very small numbers of cases (see Table 3). **D** Duration of hospital stay during pre-pandemic and pandemic years. Distribution of the length of hospital stay among all cases admitted before the pandemic (B), during year 1 of the pandemic (P1), and until 31.08 of year 2 of the pandemic (P2), for each diagnosis. P values from Kruskal–Wallis rank sum tests comparing numbers of days among the three periods are given. Note that some boxes represent very small numbers of cases

The case severity (PCCL) changed significantly for the following ICD-10 diagnosis groups during the pandemic period (Table 3, Fig. 2A, Suppl. Figure 3B): respiratory diseases (*p < *0.001) and neurologic diseases (*p = *0.022) (lower proportion of severe cases during the entire observation period), gastrointestinal diseases (*p < *0.001) (higher proportion of severe cases during the first pandemic year), perinatal conditions (*p = *0.035), and congenital malformations (*p = *0.006) (lower proportion in second pandemic year).

**Table 3:** Admissions and severity during pre-pandemic and pandemic years for major diagnostic groups

	Number of admissions			% of severe cases		
	B	P1	P2	B	P1	P2
Respiratory diseases	899	454	453	41	13	15
Non-respiratory infections	516	370	238	17	13	9
Trauma	829	1015	486	3	4	3
Psychiatric disorders	103	95	53	7	12	11
Gastrointestinal diseases	438	380	218	15	22	15
Dermatologic diseases	125	88	48	11	3	8
Malignant neoplasms	244	189	57	41	38	35
Benign neoplasms	37	23	10	17	22	10
Cardiovascular diseases	24	30	12	24	20	42
Endocrine and metabolic	73	85	25	16	21	20
Genitourinary diseases	162	159	61	21	17	23
Hematologic diseases	37	26	10	28	38	20
Musculosceletal diseases	210	203	100	33	40	40
Neurologic diseases	145	132	51	27	17	14
Ear diseases	38	24	13	16	21	15
Eye diseases	16	9	8	19	0	12
Perinatal conditions	258	238	103	42	41	28
Congenital malformations	270	260	136	31	30	19
Fungi and parasites	6	5	4	11	0	25
Poisoning, damag. impacts	24	28	11	1	0	0
Nutritional disorders	6	5	6	53	20	33
Diagnosis not specified	189	190	107	11	11	7

Total number of admissions (the 3 pre-pandemic years were calculated as an annual average (B)), percentage of severe cases (severity grades 3 or 4) and median duration of hospital stay (on days) before the pandemic (B), during the first year of the pandemic (P1), and until August of the second year of the pandemic (P2), for each group of diagnoses

During the pandemic, there were significantly longer LOS in gastrointestinal diseases (*p = *0.007), significantly shorter LOS in perinatal conditions (*p < *0.001), and congenital malformations (*p < *0.001) among all admitted cases in the ICD-10 diagnosis groups (Fig. 2B, Suppl. Figure 3C).

During pandemic, the PCCL changed significantly for RSV (*p < *0.001) and bronchiolitis (*p < *0.001) hospitalizations. For both, the proportion of severe cases decreased during the second year of the pandemic (Table 4, Fig. 2C).

**Table 4:** Total number of admissions and percentage of severe cases (severity grades 3 or 4) before the pandemic (B), during the first year of the pandemic (P1), and until 31 August of the second year of the pandemic (P2) for each diagnosis

	Number of admissions			% of severe cases		
	B	P1	P2	B	P1	P2
RSV	143	4	169	69	100	18
Bronchiolitis	141	9	108	69	22	21
Pneumonia	131	27	49	69	52	39
Influenza	43	2	0	54	0	-
Otitis	115	29	44	42	24	16
Mastoiditis	11	7	3	24	14	33
Pertussis	3	1	0	40	0	-
Preterm birth	139	100	36	64	72	53

Total number of admissions (the 3 pre-pandemic years were calculated as an annual average (B)), percentage of severe cases (severity grades 3 or 4) and median duration of hospital stay (on days) before the pandemic (B), during the first year of the pandemic (P1), and until August of the second year of the pandemic (P2), for each group of diagnoses

The LOS for the admissions with RSV (*p < *0.001), pneumonia (*p = *0.023), and preterm birth (*p = *0.007) increased during the first pandemic year followed by decreases in the second pandemic year (Fig. 2D, Suppl. Figure 3C).

## Discussion

This retrospective single-center study found significant changes in admissions in children for respiratory infections, non-respiratory and non-communicable disease when comparing seasons before and during current SARS-CoV-2 pandemic.

Our findings support the growing evidence that the pandemic and associated measures have had an impact on a wide range of infectious as well as non-infectious diseases. In a retrospective study of 42 US children's hospitals, Gill et al. found that all-cause admissions and many condition-specific admissions decreased in the spring and summer of 2020 [4]. Similar results have been shown in analyses of different cohort sizes in several countries. In particular, the decrease in communicable diseases among children during lockdowns has been highlighted in several other studies [8–12, 20].Many of these studies were limited to the first pandemic year. Our results are largely congruent, although it should be noted that our study did not show a difference in number of admissions from the baseline period for all diagnosis groups. Blocking transmission of respiratory pathogens through non-pharmacological interventions, such as masking, social distancing, and hand hygiene, likely explains the drop in inpatient stay for mainly respiratory infectious diseases. These are transmitted by droplets or aerosols, while infections with other routes of transmission were largely unaffected [3]. It is also conceivable that some parents were hesitant to take their child with mild symptoms to the hospital and rather practiced more watchful waiting.

Of note, admission for COVID-19 has not played a major role in pediatric hospitalisations in Switzerland [21]. Surprisingly but similarly to others, we identified a rapid increase in respiratory infections at the beginning of June of the second pandemic year mainly due to RSV infections including bronchiolitis. RSV usually does not occur during summer but in fall and winter [22]; however, it was completely absent in fall of 2020. Delay of the RSV season into spring 2021 could be attributable to social distancing and hygiene measures in 2020 leading to reduced exposure of the immune system. Subsequent lack of immunity might have been responsible for the increase of RSV infections after lifting restrictions and consecutive RSV exposure in spring 2021. A study investigating potential drivers for RSV rebound during the pandemic highlighted the population susceptibility as the only statistically significant driving factor for this shift in season [23]. The observed increased severity and LOS of gastrointestinal diseases during the first pandemic year might be related to the effects of changes respiratory microbiota on gut microbiota composition and trained immunity [24, 25]. In contrast, a South Korean study reported a decrease of transmissible gastrointestinal infections during the pandemic, but did not report severity [13].

Our study interestingly found a significantly higher hospitalization rate for trauma in the summer and winter months of the pandemic. Data available so far on this issue are inconclusive. A study examined trends in trauma admissions volume and injury patterns during the COVID-19 pandemic, showing a transient decrease in volume followed by a quick return to baseline levels. [14] Other studies had variable results with either lower rates or no change in admission rates. However, so far, there is consensus that the injury patterns in adults and children have changed significantly [15–18]. As previously suggested, changes in leisure behavior with an increase in trauma-prone activities such as hiking and skiing might be responsible for our findings. Remarkably, in Switzerland, the COVID-19 stringency index was consistently lower compared to neighboring countries, e.g., spending time outdoors was permitted during the entire pandemic period [26]. School closures and prolonged non-participation in sports clubs may have also contributed to a decline in movement coordination [27].

Our study has several strengths and limitations. First, we covered the first 18 months of the pandemic and included almost all (97.4%) ICD-10 main diagnoses. Second, we used a 3-year pre-pandemic reference period to account for random fluctuations. Third, in contrast to most other studies, we also evaluated duration of hospital stay and assessed severity by PCCL, which is an important addition to the literature.

In contrast, our study is limited by the design, as only the cohort of a single hospital was examined. However, being the only children’s hospital in the area, we have a large and consistent catchment population of around 180′000 children and adolescents [28–31]. Nevertheless, as some diagnoses were rare, they could not be meaningfully recorded individually and were therefore subsumed under the bigger diagnosis groups.

In conclusion, our study shows that the COVID-19 pandemic strongly affects the number and distribution of pediatric hospital admissions. Whereas many diagnosis groups, especially some respiratory and non-respiratory infections, were significantly lower during the first pandemic year, trauma was the only diagnosis group with an increase. For some respiratory infections an opposite trend was evidenced at the end of the first 18 months of the pandemic. Severity indicators were lower for most diagnoses including respiratory infections. It remains to be seen whether these trends continue during the subsequent course of the pandemic and beyond.

## Supplementary Information

Below is the link to the electronic supplementary material.

**Supplementary file 1**: Suppl. Figure 1A Confirmed COVID-19 cases in Switzerland March 2020 to August 2021; CHE –Switzerland, whole country; grR - greater region; vertical axis: new cases. Suppl. Figure 1B Oxford Stringency Index for Switzerland, fully vaccinated people in Switzerland and % mobility change (car driving) from March 2020 to August 2021. Figures were created using the following online tool: ethz. COVID-19 Re (ETH Zürich, Switzerland), https://ibz-shiny.ethz.ch/covid-19-re-international/ (Accessed 5.11.2021).

**Supplementary file 2**: Suppl. Figure 2 Occupancy rates at the Children's Hospital of eastern Switzerland in % from March 2017 to August 2021

**Supplementary file 3**: Suppl. Figure 3A Weekly number of admissions from 21 March 2017 to 31 August 2021; Number of admissions (points) and smoothing lines obtained with local polynomial regression (loess) separately for the three years preceding the pandemic and during the pandemic. To represent the entire time course of the pandemic by one line, the time axis ranges from 21 March 2020 to 31 August 2021. For the three years preceding the pandemic, weeks from 21 March to 31 August are duplicated at the end of the time axis (open symbols). P-values from Poisson models for the overall difference between the two periods (regardless of season) are given. Suppl. Figure 3B Distribution of case severity (ranked from 0 to 4) among all cases admitted before the pandemic (B), during the first year of the pandemic (P1), and until August of the second year of the pandemic (P2) for each group of diagnoses. Suppl. Figure 3C Distribution of the length of hospital stay among all cases admitted before the pandemic (B), during the first year of the pandemic (P1), and until August of the second year of the pandemic (P2) for each group of diagnoses. P-values Suppl. Figures 3B/C from Kruskal-Wallis rank sum tests comparing severity grades between the three periods are given.

**Supplementary file 4**: **Table S1 **Overview of the groups of diagnoses with short name for figures / tables and number of cases (admissions pre-pandemic and pandemic years). **Table S2 **Rate ratios for changes in the mean number of admissions from pre-pandemic to pandemic years for major diagnostic groups. **Table S3 **Effects of pandemic and season on weekly number of hospital admissions for major respiratory diagnoses during the first and second pandemic years.
