# Supplementary figures and images for: Pediatric hospital admissions, case severity, and length of hospital stay during the first 18 months of the COVID-19 pandemic in a tertiary children’s hospital in Switzerland
Source: Infection. 2022 Sep 5;51(2):439–46. doi: 10.1007/s15010-022-01911-x (PMC9444086; doi:10.1007/s15010-022-01911-x)

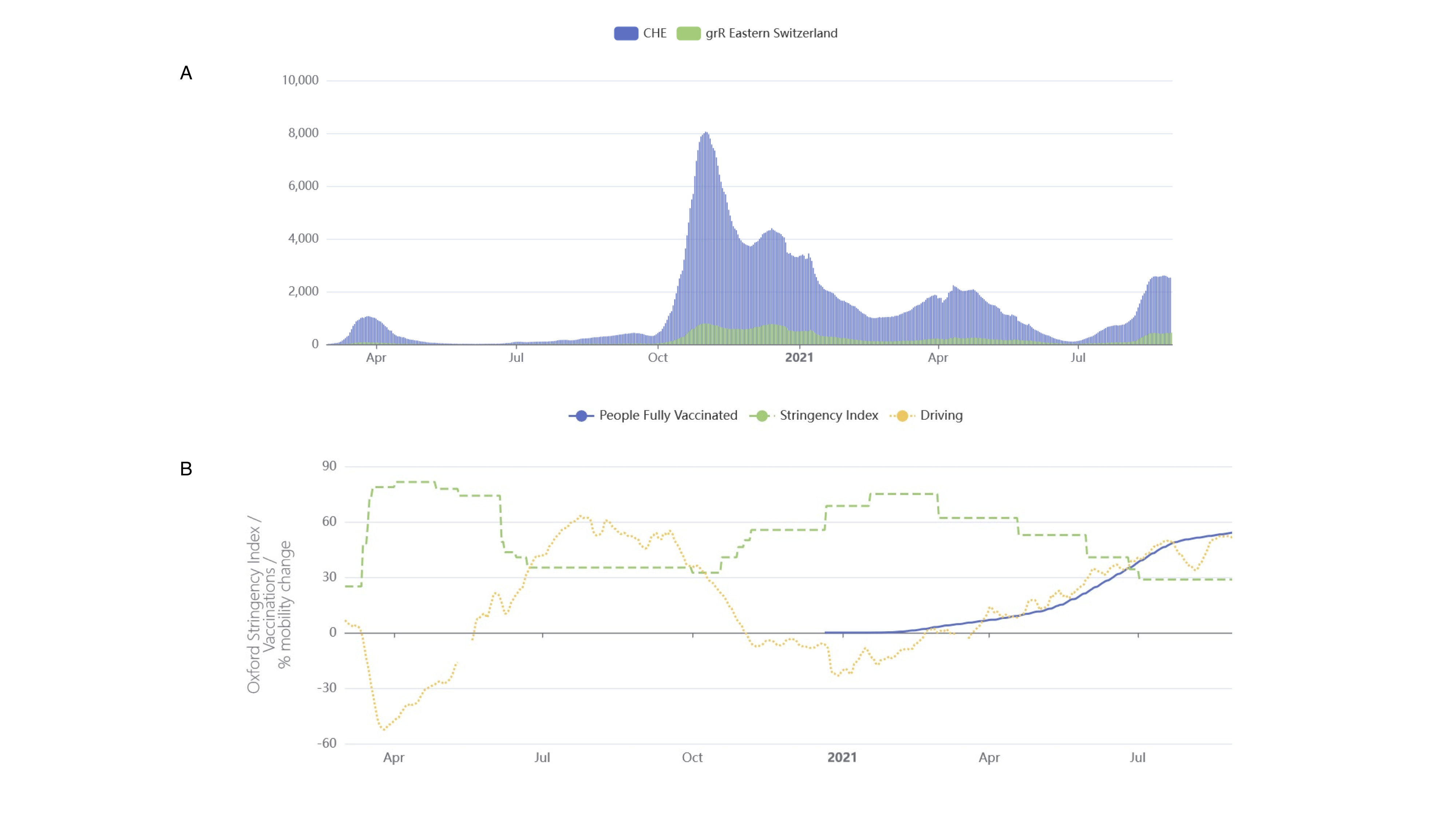

Supplement: Supplementary file 1 — Suppl. Figure 1A Confirmed COVID-19 cases in Switzerland March 2020 to August 2021; CHE –Switzerland, whole country; grR - greater region; vertical axis: new cases. Suppl. Figure 1B Oxford Stringency Index for Switzerland, fully vaccinated people in Switzerland and % mobility change (car driving) from March 2020 to August 2021. Figures were created using the following online tool: ethz. COVID-19 Re (ETH Zürich, Switzerland), https://ibz-shiny.ethz.ch/covid-19-re-international/ (Accessed 5.11.2021). [file 15010_2022_1911_MOESM1_ESM.png]

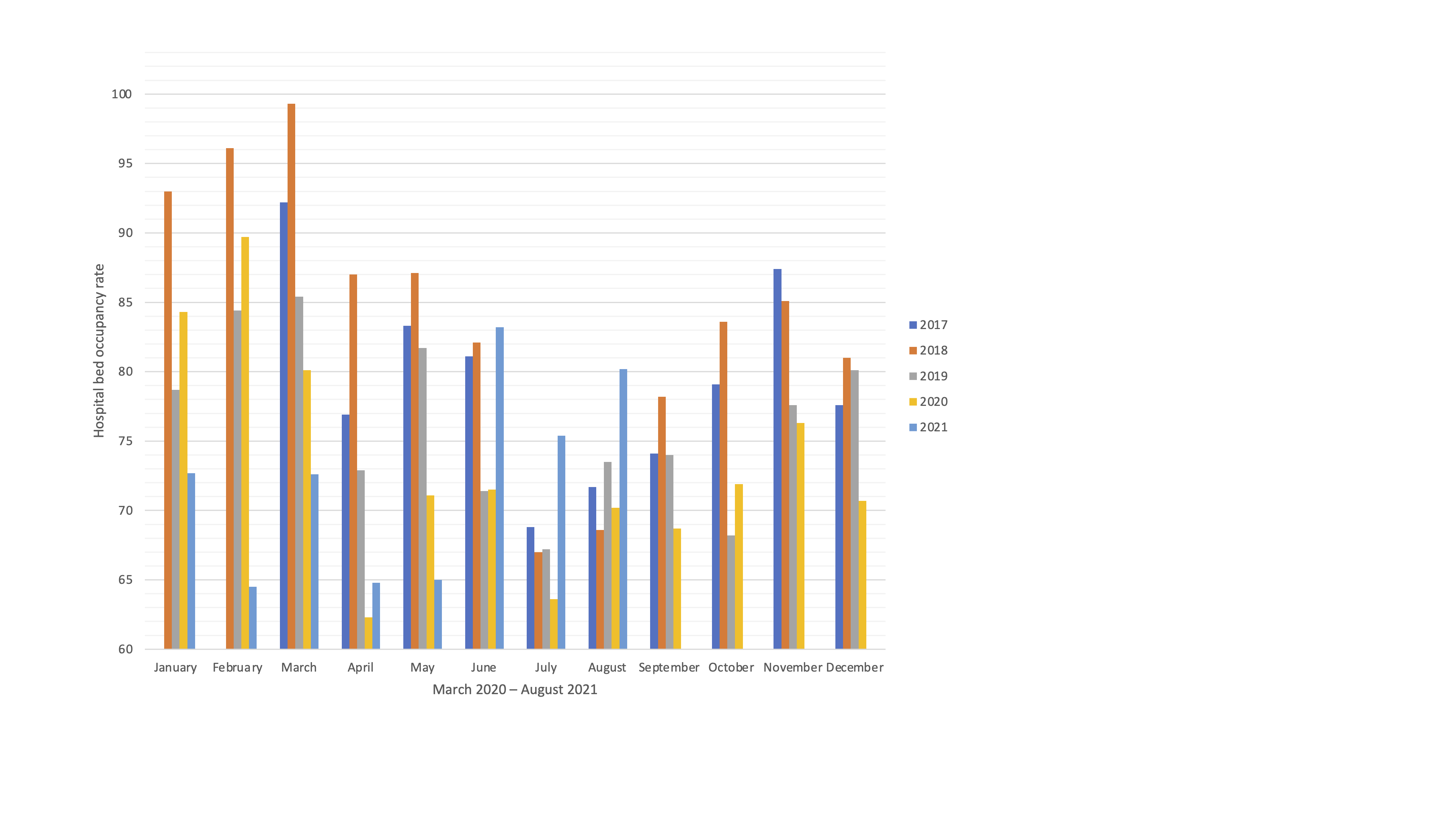

Supplement: Supplementary file 2 — Suppl. Figure 2 Occupancy rates at the Children's Hospital of eastern Switzerland in % from March 2017 to August 2021 [file 15010_2022_1911_MOESM2_ESM.png]

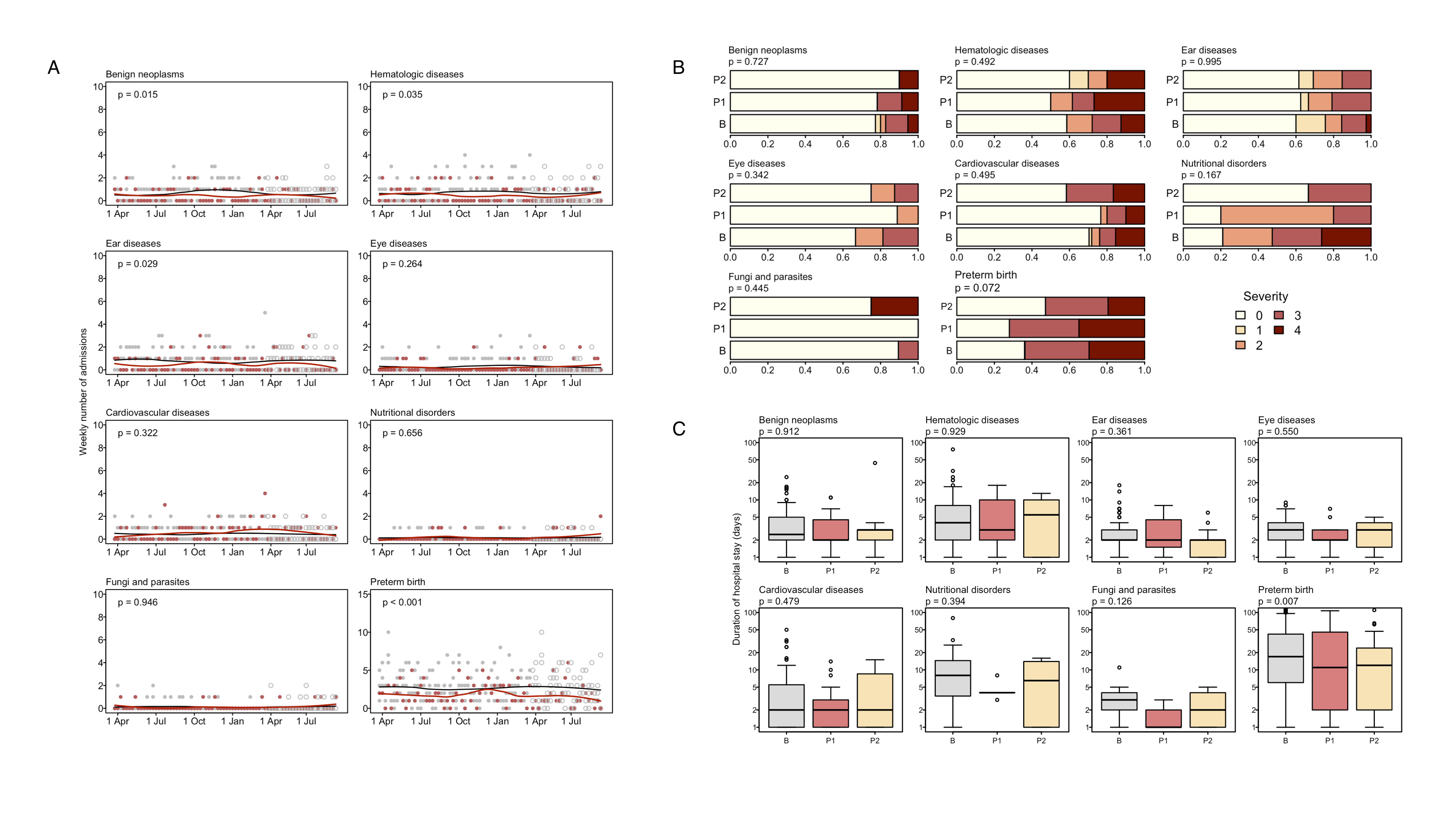

Supplement: Supplementary file 3 — Suppl. Figure 3A Weekly number of admissions from 21 March 2017 to 31 August 2021; Number of admissions (points) and smoothing lines obtained with local polynomial regression (loess) separately for the three years preceding the pandemic and during the pandemic. To represent the entire time course of the pandemic by one line, the time axis ranges from 21 March 2020 to 31 August 2021. For the three years preceding the pandemic, weeks from 21 March to 31 August are duplicated at the end of the time axis (open symbols). P-values from Poisson models for the overall difference between the two periods (regardless of season) are given. Suppl. Figure 3B Distribution of case severity (ranked from 0 to 4) among all cases admitted before the pandemic (B), during the first year of the pandemic (P1), and until August of the second year of the pandemic (P2) for each group of diagnoses. Suppl. Figure 3C Distribution of the length of hospital stay among all cases admitted before the pandemic (B), during the first year of the pandemic (P1), and until August of the second year of the pandemic (P2) for each group of diagnoses. P-values Suppl. Figures 3B/C from Kruskal-Wallis rank sum tests comparing severity grades between the three periods are given. [file 15010_2022_1911_MOESM3_ESM.png]
