# Supplementary material for: Pediatric hospital admissions, case severity, and length of hospital stay during the first 18 months of the COVID-19 pandemic in a tertiary children’s hospital in Switzerland
Source: Infection. 2022 Sep 5;51(2):439–46. doi: 10.1007/s15010-022-01911-x (PMC9444086; doi:10.1007/s15010-022-01911-x)
Supplement: Supplementary file 4 — Table S1 Overview of the groups of diagnoses with short name for figures / tables and number of cases (admissions pre-pandemic and pandemic years). Table S2 Rate ratios for changes in the mean number of admissions from pre-pandemic to pandemic years for major diagnostic groups. Table S3 Effects of pandemic and season on weekly number of hospital admissions for major respiratory diagnoses during the first and second pandemic years. [file 15010_2022_1911_MOESM4_ESM.docx]

**Suppl. Table 1** Overview of the groups of diagnoses with short name for figures / tables and number of cases (admissions pre-pandemic and pandemic years)

| Group name | Short name | Cases |
| --- | --- | --- |
| Respiratory diseases | Respiratory diseases | 3603 |
| Nonrespiratory diseases | Nonrespiratory infections | 2157 |
| Trauma | Trauma | 3988 |
| Mental and behavioural disorders | Psychiatric disorders | 456 |
| Diseases of the digestive system | Gastrointestinal diseases | 1913 |
| Diseases of the skin and subcutaneous tissue | Dermatologic diseases | 512 |
| Malignant neoplasms | Malignant neoplasms | 977 |
| Benign neoplasms | Benign neoplasms | 143 |
| Diseases of the circulatory system | Cardiovascular diseases | 113 |
| Endocrine and metabolic disorders | Endocrine and metabolic disorders | 329 |
| Diseases of the genitourinary system | Genitourinary diseases | 706 |
| Diseases of the blood and blood-forming organs and certain disorders involving the immune mechanism | Hematologic diseases | 147 |
| Diseases of the musculosceletal system and connective tissue | Musculosceletal diseases | 934 |
| Diseases of the nervous system | Neurologic diseases | 618 |
| Diseases of the ear and mastoid process | Ear diseases | 152 |
| Diseases of the eye and adnexa | Eye diseases | 65 |
| Certain conditions originating in the perinatal period | Perinatal conditions | 1116 |
| Congenital malformations | Congenital malformations | 1206 |
| Fungi and parasites | Fungi and parasites | 28 |
| Poisoning and certain other consequences of external causes | Poisoning, damaging impacts | 111 |
| Nutritional disorders | Nutritional disorders | 30 |
| Symptoms, signs and abnormal clinical and laboratory findings, not elsewhere classified | Diagnosis not specified | 864 |

Disease groups were subdivided into (1) those expected or found to change significantly during the pandemic, (2) other general medical diagnoses, (3) perinatal, rare and unspecified diagnoses.

**Suppl. Table 2** Rate ratios for changes in the mean number of admissions from pre-pandemic to pandemic years for major diagnostic groups

**Year 1**

| Group | spring | summer | autumn | winter |
| --- | --- | --- | --- | --- |
| Respiratory diseases | 0.4 (0.3 to 0.5) | 1.0 (0.9 to 1.3) | 0.6 (0.5 to 0.8) | 0.3 (0.3 to 0.4) |
| Nonrespiratory infections | 0.6 (0.5 to 0.8) | 0.8 (0.6 to 0.9) | 0.8 (0.7 to 1.0) | 0.7 (0.5 to 0.8) |
| Trauma | 1.0 (0.9 to 1.2) | 1.4 (1.2 to 1.6) | 1.2 (1.0 to 1.4) | 1.3 (1.2 to 1.5) |
| Psychiatric disorders | 0.6 (0.3 to 1.0) | 1.0 (0.6 to 1.6) | 1.1 (0.7 to 1.6) | 1.1 (0.7 to 1.7) |
| Gastrointestinal diseases | 0.8 (0.7 to 1.0) | 0.9 (0.7 to 1.1) | 1.0 (0.8 to 1.3) | 0.8 (0.6 to 1.0) |
| Dermatologic diseases | 0.7 (0.4 to 1.2) | 0.8 (0.5 to 1.2) | 0.8 (0.5 to 1.3) | 0.5 (0.3 to 0.8) |
| Malignant neoplasms | 0.6 (0.4 to 0.8) | 0.7 (0.5 to 1.0) | 0.9 (0.6 to 1.2) | 1.0 (0.8 to 1.4) |
| Benign neoplasms | 0.8 (0.3 to 1.9) | 0.9 (0.4 to 2.1) | 0.5 (0.2 to 1.1) | 0.6 (0.2 to 1.4) |
| Cardiovascular diseases | 0.5 (0.2 to 1.6) | 1.5 (0.7 to 3.5) | 1.1 (0.4 to 2.7) | 1.8 (0.9 to 3.5) |
| Endocrine and metabolic disorders | 1.3 (0.8 to 2.1) | 1.5 (0.9 to 2.5) | 0.9 (0.6 to 1.5) | 1.0 (0.6 to 1.6) |
| Genitourinary diseases | 1.3 (1.0 to 1.8) | 0.7 (0.5 to 1.1) | 0.9 (0.7 to 1.3) | 1.0 (0.7 to 1.4) |
| Hematologic diseases | 1.1 (0.5 to 2.5) | 1.1 (0.5 to 2.2) | 0.4 (0.1 to 1.0) | 0.5 (0.2 to 1.2) |
| Musculosceletal diseases | 1.0 (0.7 to 1.4) | 1.2 (0.8 to 1.6) | 0.9 (0.6 to 1.2) | 0.9 (0.6 to 1.2) |
| Neurologic diseases | 1.1 (0.7 to 1.6) | 0.9 (0.6 to 1.3) | 1.0 (0.7 to 1.4) | 0.7 (0.5 to 1.1) |
| Ear diseases | 0.4 (0.2 to 1.1) | 0.5 (0.2 to 1.2) | 1.1 (0.6 to 2.3) | 0.6 (0.2 to 1.6) |
| Eye diseases | 0.8 (0.2 to 2.7) | 0.8 (0.2 to 3.1) | 0.4 (0.1 to 1.7) | 0.5 (0.1 to 1.8) |
| Perinatal conditions | 1.0 (0.7 to 1.3) | 0.9 (0.7 to 1.2) | 1.0 (0.8 to 1.4) | 0.8 (0.6 to 1.1) |
| Congenital malformations | 0.7 (0.5 to 0.9) | 1.0 (0.7 to 1.4) | 1.2 (1.0 to 1.5) | 1.0 (0.7 to 1.3) |
| Fungi and parasites | 1.3 (0.3 to 6.0) | 0.1 (0.0 to 2.1) | 1.1 (0.2 to 4.8) | 1.2 (0.2 to 8.6) |
| Poisoning, other damaging impacts | 1.3 (0.4 to 3.8) | 1.4 (0.8 to 2.6) | 1.3 (0.5 to 3.0) | 0.4 (0.1 to 1.6) |
| Nutritional disorders | 0.3 (0.0 to 4.9) | 1.2 (0.3 to 4.2) | 0.8 (0.1 to 4.7) | 0.6 (0.1 to 3.8) |
| Diagnosis not specified | 0.8 (0.5 to 1.1) | 0.9 (0.6 to 1.3) | 1.0 (0.8 to 1.4) | 1.4 (1.0 to 1.8) |

**Suppl. Table 2 (cont. ) Year 2**

| Group | spring | summer |
| --- | --- | --- |
| Respiratory diseases | 0.9 (0.8 to 1.1) | 2.7 (2.3 to 3.2) |
| Nonrespiratory infections | 0.8 (0.7 to 1.1) | 1.1 (0.9 to 1.3) |
| Trauma | 1.3 (1.2 to 1.6) | 1.1 (1.0 to 1.3) |
| Psychiatric disorders | 1.2 (0.7 to 1.8) | 1.2 (0.8 to 1.9) |
| Gastrointestinal diseases | 0.9 (0.7 to 1.2) | 1.2 (1.0 to 1.5) |
| Dermatologic diseases | 0.8 (0.5 to 1.4) | 0.9 (0.6 to 1.3) |
| Malignant neoplasms | 0.5 (0.4 to 0.8) | 0.4 (0.3 to 0.6) |
| Benign neoplasms | 1.1 (0.5 to 2.7) | 0.5 (0.2 to 1.4) |
| Cardiovascular diseases | 1.1 (0.4 to 2.7) | 1.0 (0.4 to 2.6) |
| Endocrine and metabolic disorders | 0.8 (0.4 to 1.5) | 0.7 (0.4 to 1.3) |
| Genitourinary diseases | 0.8 (0.6 to 1.3) | 0.8 (0.6 to 1.2) |
| Hematologic diseases | 0.7 (0.2 to 2.3) | 0.9 (0.4 to 2.0) |
| Musculosceletal diseases | 1.1 (0.8 to 1.5) | 1.1 (0.8 to 1.6) |
| Neurologic diseases | 1.2 (0.8 to 1.8) | 0.6 (0.4 to 0.9) |
| Ear diseases | 0.6 (0.2 to 1.3) | 0.8 (0.3 to 1.7) |
| Eye diseases | 0.8 (0.2 to 2.7) | 2.5 (0.8 to 8.0) |
| Perinatal conditions | 1.0 (0.7 to 1.3) | 0.8 (0.6 to 1.1) |
| Congenital malformations | 1.1 (0.8 to 1.4) | 1.4 (1.0 to 1.8) |
| Fungi and parasites | 0.9 (0.1 to 6.1) | 1.2 (0.3 to 4.2) |
| Poisoning, other damaging impacts | 2.3 (0.8 to 6.4) | 0.6 (0.2 to 1.4) |
| Nutritional disorders | 1.6 (0.3 to 7.8) | 1.0 (0.2 to 4.0) |
| Diagnosis not specified | 1.3 (0.9 to 1.8) | 1.2 (0.9 to 1.6) |

Rate ratios for changes in the mean number of admissions in each season due to the pandemic with 95% CI, derived from Bayesian Poisson regression with log link.

Most of the 95% CI include a ratio of 1, meaning that the existence of an increase or decrease between the two periods *in a particular season* cannot be generalized with 95% confidence.

Blue colour indicates decreased numbers in a season of the pandemic (rounded upper limit of the CI < 1.0), red colour indicates increased numbers (rounded lower limit of the CI > 1.0). This subdivision is only descriptive and not based on formal tests of significance.

**Suppl. Table 3** Effects of pandemic and season on weekly number of hospital admissions for major respiratory diagnoses during the first and second pandemic years.

|  | Year 1 of the pandemic | | |  | Year 2 of the pandemic | | |
| --- | --- | --- | --- | --- | --- | --- | --- |
|  | COVID-19 | season | COVID-19*season |  | COVID-19 | season | COVID-19*season |
| RSV | <0.001 | <0.001 | 0.001 |  | <0.001 | <0.001 | <0.001 |
| Bronchiolitis | <0.001 | <0.001 | 0.003 |  | <0.001 | 0.027 | <0.001 |
| Pneumonia | <0.001 | <0.001 | 0.064 |  | 0.054 | 0.966 | <0.001 |
| Influenza | <0.001 | <0.001 | 0.011 |  | 0.090 | 0.138 | 1 |
| Otitis | <0.001 | <0.001 | 0.089 |  | 0.892 | <0.001 | 0.003 |
| Mastoiditis | 0.255 | 0.687 | 0.024 |  | 0.275 | 0.800 | 0.722 |
| Pertussis | 0.177 | 0.808 | 0.664 |  | 0.283 | 0.951 | 1 |
| Preterm birth | 0.002 | 0.513 | 0.367 |  | 0.001 | 0.098 | 0.246 |

*P*-values from two-way analysis of deviance for the effects of COVID-19, season and the interaction on weekly hospital admissions during the first year (until March 2021) and second year (until August 2021) of the pandemic. The effect of COVID-19 was determined as the difference in mean weekly numbers between the three pre-pandemic years and one year of the pandemic. Seasons were defined as four 13-weeks periods for the analysis of Year 1 and two 11/12-week periods for the analysis of Year 2.
